# Aggressive Giant Cell Reparative Granuloma of the Nasal Cavity

**DOI:** 10.1155/2013/690194

**Published:** 2013-03-27

**Authors:** Hajime Ishinaga, Kazuya Otsu, Genshin Mouri, Kazuhiko Takeuchi

**Affiliations:** ^1^Department of Otorhinolaryngology-Head & Neck Surgery, Mie University Graduate School of Medicine, 2-174 Edobashi, Mie, Tsu 514-8507, Japan; ^2^Department of Neurosurgery, Mie University Graduate School of Medicine, Tsu 514-8507, Japan

## Abstract

Giant cell reparative granuloma (GCRG) is an uncommon and nonneoplastic reactive tumor that involves the maxilla and mandible in the region of the head and neck. It is rare in the nasal cavity, and it might be misdiagnosed. We reported a very aggressive GCRG with intracranial invasion, which was treated surgically via a combined approach of a lateral rhinotomy with a craniotomy by bilateral coronal incision. The pathology was consistent with GCRG. A short literature review about diagnosis, clinical behavior, and treatment of this tumor entity is given.

## 1. Introduction

The term giant cell reparative granuloma (GCRG) was first introduced by Jaffe in 1953 [1] and is an uncommon nonneoplastic reactive tumor that occurs almost exclusively within the mandible and maxilla. The second most common location is in the bone of the hands and feet. The etiology of GCRG is uncertain but may be related to an intraosseous hemorrhage following trauma. In addition, microscopic evidence of hemorrhagic cyst formation resembling an aneurysmal bone cyst frequently is present [2]. GCRGs can be observed at all ages, and they tend to appear more often in children and young adults. Despite their benign nature, they may be locally aggressive. Only a small number of cases have been reported with the involvement of the paranasal sinuses and the orbit [3, 4]. In this paper, we report a new case of GCRG originated from nasal cavity with intracranial invasion.

## 2. Case Report

A 49-year-old women was admitted to the department of otorhinolaryngology in an affiliated hospital with a 2-month history of right-sided nasal obstruction, epistaxis, proptosis, and diplopia. The mass was biopsied endoscopically. Massive bleeding from the tumor was seen at that time. Histopathological diagnosis was reparative giant cell granuloma. Oral steroids (30 mg prednisolone) were administered due to rapid growth of the tumor for two weeks. Since the treatment was not effective, the patient was sent to our hospital. 

She was a nonsmoker, and there was no history of facial trauma and previous radiation exposure. Her past medical history was unremarkable, and there was no significant family history. Physical examination revealed that the mass completely obstructed the right nasal cavity and partially the left side. The outer surface of the mass was smooth and displaced the right eyeball towards the lateral side, and consequently led to the diplopia. There were no palpable masses or lymphadenopathies in the neck. Routine hematological and biochemical test results including serum calcium, alkaline phosphatase, phosphorus, renal function, and parathyroid hormone were within normal limits.

**Figure 1:**
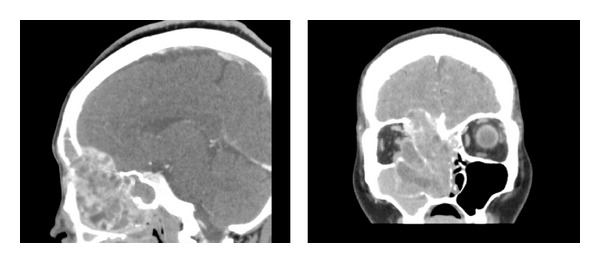
Preoperative computed tomography (contrast-enhanced) demonstrates the mass occupied right nasal cavity with intracranial invasion and extension into the orbit.

Computerized tomography (CT) scanning showed expansile mass and with a multilocular appearance and extension into the orbit, ethmoid sinus, maxillary sinus, and intracranial region (Figure 1).

**Figure 2:**
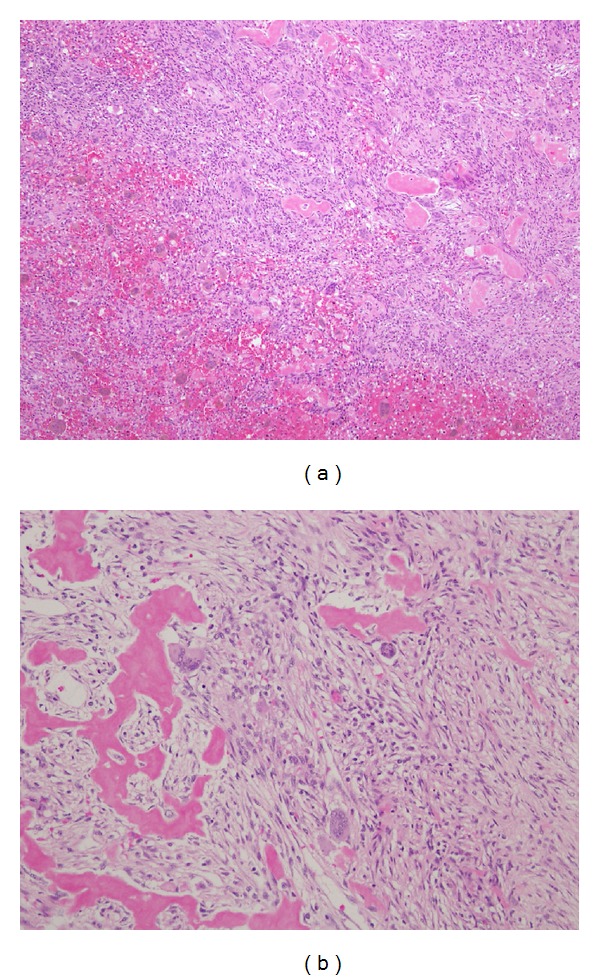
(a) (hematoxylin eosin, original magnification ×20) Granulomatous tissue with numerous characteristic multinucleate giant cells and a large quantity of mononuclear infiltrative cells. (b) (hematoxylin eosin, original magnification ×100) Histopathological examination showed a richly cellular fibroblastic proliferation with osteoclast-like polynuclear giant cells interspersed with spindle-shaped stroma, spicules of newly formed bone.

**Figure 3:**
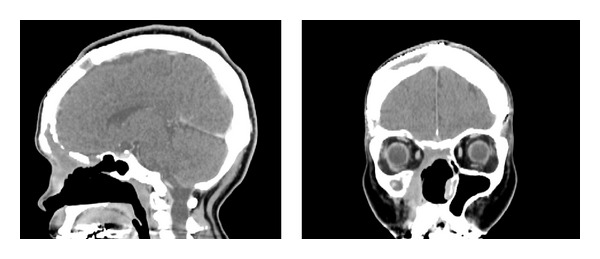
Postoperative computed tomography (contrast-enhanced) shows no recurrence that is seen in both nasal cavities and the intracranial space.

The patient underwent surgical resection via combined approach of a lateral rhinotomy with a craniotomy by bilateral coronal incision after bilateral maxillary artery embolism. The mass originated from the localization of the nasal cavity and eroded lamina papyracea, medial wall of maxilla, and anterior skull base. There was strong adhesion to the dura and the medial rectus; therefore, the mass was excised en bloc except above lesion, and then we performed additional resection for complete surgical excision. Skull base reconstruction was done by cranial bone and pericranial flap. Definitive histologic examination revealed a fibroblastic proliferation with rich osteoclast-like polynuclear giant cells interspersed with spindle-shaped stroma. (Figures 2(a) and 2(b)). There were no enophthalmus and diplopia in the postoperative period, and the patient's postoperative course was uneventful. The patient was free of recurrence after 1 year of followup (Figure 3).

## 3. Discussion

 Giant cell reparative granuloma most commonly involved the mandible. To our knowledge, this is the first report of GCRG from nasal cavity with intracranial invasion. GCRG of the craniofacial bones has to be distinguished from aneurismal bone cyst, giant cell tumor, and Brown tumor. Aneurismal bone cyst usually arises from the vertebrae and the long bones, not the facial bone. The histological differential diagnosis is accomplished with the absence of both the chondroid matrix and the new bone formation in case of giant cell tumor. In addition, a true giant cell tumor shows more giant cells and mitotic activity. Furthermore, Ca, P, and PTH levels are in normal limits in GCRG, and it can be differentiated form Brown tumor of hyperparathyroidism.

 GCRG has two clinical forms: central-endosteal and peripheral-soft tissue forms [5]. The peripheral type which involves gingival and alveolar mucosa, is encountered in women below 30 years of age, and the central type can be seen in all ages; however, it is usually encountered between the ages of 10 and 20 years and with the involvement of the mandible [6]. There is no histological difference between central and peripheral giant cell reparative granulomas. GCRG is also divided into two categories according to clinical behavior: aggressive and nonaggressive [6]. The nonaggressive form is that more commonly seen with characteristic slow-growth and painless swelling. Meanwhile, in the aggressive form, the swelling is painful and grows rapidly, and in addition, recurrence rate is high. Our case was categorized as the central type and aggressive type according to the above criteria.

 The treatment of GCRG is usually surgical. The common proposal for the treatment is surgical excision or curettage and 80% of the cases can be cured with these modalities. It may recur after incomplete removal in 10% to 15% [5]. Chuong et al. recommend the use of block resection in aggressive lesion cases that show painful, cortical bone perforation [7]. The recurrence rate has been defined between 11% and 35% in the literature [8]. In our case, the tumor showed tight adhesion with the dura and the medial rectus; therefore, it was difficult to perform en bloc resection without causing the functional disturbance. Consequently, surgical excision and additional curettage method were performed in the present case without any signs of recurrence.

 Because of the possibility of recurrence especially in aggressive type, it should be acquainted with the alternative treatment of GCRG. The use of steroid has been reported [9]. It is speculated that the extracellular production of bone-resorption-mediating lysosomal proteases by giant cells is inhibited by steroids which also induce apoptosis of the osteoclast-like cells [9]. Several authors have described that intralesional administration of corticosteroids showed favourable results. In our case, oral steroid administration was performed preoperatively; however, the effect was insufficient to suppression of the tumor growth. Different studies showed a variable responses of patients to calcitonin therapy, and alpha-IFN administered for aggressive CGCGs seems capable of stopping rapid growth of the lesion and consolidating or even diminishing their size but it is still necessary to use additional surgery to eliminate the lesion. Another treatment alternative is radiotherapy, which may be suggested in the case of recurrence or unresectable tumor; however, it should be noted that some cases are radioresistant and sarcomatous transformation may take place in the long term. Furthermore, Yoshida et al. have reported that two GCRG patients were treated by curettage followed by adjuvant therapy comprising phenol and ethanol and bone grafting, and no recurrence was seen after this treatment [10].

 In conclusion, we have reported a case of aggressive GCRG originated nasal cavity with intracranial invasion. We have to put the high priority on the complete resection of GCRG, considering other alternative methods.
